# Extracellular vesicles regulate the transmission of insulin resistance and redefine noncommunicable diseases

**DOI:** 10.3389/fmolb.2022.1024786

**Published:** 2023-01-09

**Authors:** Biao Li, Wan Li, Tiancai Liu, Longying Zha

**Affiliations:** ^1^ Department of Nutrition and Food Hygiene, Guangdong Provincial Key Laboratory of Tropical Disease Research, National Medical Products Administration Key Laboratory of Cosmetic Safety Evaluation, School of Public Health, Southern Medical University, Guangzhou, Guangdong, China; ^2^ School of Physical Education, Hubei Minzu University, Enshi, China; ^3^ Key Laboratory of Antibody Engineering of Guangdong Higher Education Institutes, School of Laboratory Medicine and Biotechnology, Southern Medical University, Guangzhou, Guangdong, China

**Keywords:** insulin resistance, extracellular vesicle, insulin receptor substrate, T2DM, diabetes

## Abstract

Noncommunicable diseases (NCDs), such as diabetes and related neurological disorders, are considered to not be directly transmissible from one person to another. However, NCDs may be transmissible *in vivo* through extracellular vesicles (EVs). A long-term high-fat diet (HFD) can induce a series of health issues like hyperlipidemia, type 2 diabetes mellitus (T2DM), and diabetic peripheral neuropathy (DPN) due to insulin resistance. Multiple molecular signaling changes can stimulate insulin resistance, especially blocking insulin signaling by increased insulin resistance inducer (phosphorylation of negative regulatory sites of insulin receptor substrate (IRS) proteins) and decreased tyrosine phosphorylation of insulin receptor substrate (phosphorylation of positive regulatory sites of IRS), thus leading to reduced phosphorylation of AKT enzymes. Current efforts to treat T2DM and prevent its complications mainly focus on improving insulin sensitivity, enhancing insulin secretion, or supplementing exogenous insulin based on a common assumption that insulin resistance is noncommunicable. However, insulin resistance is transmissible within multiple tissues or organs throughout the body. Exploring the regulatory roles of EVs in developing insulin resistance may provide novel and effective preventive and therapeutic strategies.

## Introduction

The balance between energy consumption and intake is crucial to human health. Excessive energy intake can result in multiple metabolic diseases, including type 2 diabetes mellitus (T2DM) (Anderson et al., 2003), due to high-level blood glucose, deficient insulin secretion, and insulin resistance. Insulin resistance also can be induced by multiple factors, such as heredity, aging, and inflammation. The progression from insulin resistance to T2DM may take decades. Current treatments of insulin resistance or T2DM focus on promoting insulin sensitivity, improving islet *β*-cell function to enhance insulin secretion, or supplementing exogenous insulin. It is generally recognized that obesity can develop into T2DM with a long-term process of chronic excessive energy intake–obesity–insulin resistance–T2DM (Weyer et al., 1999; Leitner et al., 2017). The transmission of insulin resistance is also involved and critical in the development course of T2DM (Wang et al., 2017; Ying et al., 2017). Long-term hyperglycemia can induce chronic debilitating complications due to the toxicity of high-level glucose (Van den Berghe, 2004). Therefore, maintaining blood glucose levels is crucial to health. Usually, obesity is a common inducer of early T2DM, resulting from high caloric consumption and an irregular diet. Binge eating disorder is characterized by repeated gluttony, leading to a high incidence of chronic hyperglycemia among early diabetic patients (Allison et al., 2007; Abbott et al., 2018). To protect the body from glucose toxicity, the pancreas can produce a large amount of insulin to reduce blood glucose levels. A vicious cycle resulting from binge eating can lead to hyperinsulinemia and high serum insulin levels can cause a sense of hunger, thereby promoting the generation and accumulation of adipose tissue. During this process, insulin resistance gradually develops, as initially reflected in impaired glucose tolerance, then relative insulin deficiency, and eventually fasting hyperglycemia (Weyer et al., 1999). However, the exact mechanism for transmitting insulin resistance from tissues and organs to the whole body is still unclear. Recent evidence suggests that the factors for suppressing insulin signaling can be transmitted between cells *via* extracellular vesicles (EVs), thereby changing the definition of insulin resistance from a noncommunicable preclinical health condition to a transmissible pathological status (Dibaba et al., 2017; Ying et al., 2017).

EVs are particles delimited by a lipid bilayer and naturally released by cells (Théry et al., 2018). EVs can be classified into two major categories: microvesicles and exosomes. Microvesicles are formed by the budding of the plasma membrane, with particle sizes ranging from 50 nm to 1,000 nm. Exosomes are released from multivesicular endosomes (MVEs) after fusion with the plasma membrane. Functional studies of EVs have been spurred by their ability to transport various types of biomolecules, such as RNA, proteins, and DNA, to their recipient cells (Kapogiannis et al., 2015). Due to the similar biochemical properties and the overlapping size, it is often difficult to distinguish the functions of EVs (Kowal et al., 2016; Jeppesen et al., 2019). Currently, there is a lack of purification methods for the quantitative separation of various EV subclasses, making the assignment of functional properties to specific EV subtypes challenging. Therefore, there are no systematic studies on the roles and corresponding mechanisms of EVs in the development and transmission of insulin resistance. Recent studies have preliminarily confirmed that EVs can regulate the progression of insulin resistance (Choi et al., 2015). EVs from different cells may have opposite effects due to their different cargoes. Neutral ceramidase-enriched exosomes can prevent insulin resistance induced by palmitic acid, while the injection of exosomes from obese mice into healthy mice can lead to the development of insulin resistance (Deng et al., 2009; Zhu et al., 2016). Hence, if insulin resistance is treated as a transmissible pathological condition that can spread from cell to cell, the progression of T2DM could be inhibited by blocking its transmission.

In this article, we discuss the mechanisms that drive cell–EV–cell axis formation, thus causing insulin resistance, a point that has rarely been discussed in previous studies. Various factors may lead to insulin resistance, such as phosphorylation of most serine sites of insulin receptor substrate (IRS) proteins. It has been demonstrated that the phosphorylation of different serine residues has the opposite effect on insulin signaling (Copps and White, 2012). Here, we aim to discuss the function of the cell–EV–cell axis in the development of noncommunicable diseases (NCDs). Thus, all the “p-s-IRS” in this paper represent the phosphorylation of negative regulatory sites of IRS proteins. The word “exosome” has been used in the article, but it is noted that the term small extracellular vesicle (EV) is more precise and consistent with the latest findings in the EV field.

## Insulin resistance can be transmitted *via* the cell–EV–cell axis to protect against stress-induced cellular damage

### Insulin resistance is a cellular protective mechanism against glucose-induced ROS

The lesser sensitivity or complete nonresponsivity of cells to insulin is termed insulin resistance, a typical symptom at the early stage of T2DM. Insulin resistance can occur in multiple tissues and organs throughout the body and can be induced by a variety of inducers such as inflammation and obesity. Physiological stress can lead to a variety of cell dysfunctions, and some of these changes are protective mechanisms for improving cell survival through insulin resistance (Ye, 2013). Chronic exposure to hyperglycemia can lead to cellular dysfunction, termed glucotoxicity, which may become irreversible over time (Robertson et al., 2003; Staels, 2017). In addition to being an energy source, blood glucose can also result in side effects due to its toxicity. Glucose toxicity is reflected in its capacity to induce protein glycosylation (Burd, 2019). Glucose as a polyhydroxy aldehyde can react with amino residues of proteins to form fructosamine bonds and eventually become advanced glycation end products (AGEs) after a series of reactions. AGEs are associated with various diabetic complications such as diabetic retinopathy, kidney disease, and neurological diseases (Beisswenger, 2012). Therefore, the body can execute corresponding responses immediately by secreting insulin to reduce blood glucose levels and protect the body from glucose toxicity upon the high-level glucose stimulation.

Glucose is metabolized in cells into glyceric acid, glyceraldehyde, and acetone, thereby subsequently entering the tricarboxylic acid cycle and generating ATP through oxidative phosphorylation to provide energy for various physiological activities of cells. However, when a large amount of glucose enters cells, glycolysis is not able to consume enough glucose, and the glyceraldehyde metabolism is inhibited, thus eventually leading to the activation of nicotinamide adenine dinucleotide phosphate (NADPH) oxidases (NOX) (Han et al., 2012). NOX is a membrane protein widely distributed in tissues and organs of the body and includes multiple isoforms, such as NOX1, NOX2, NOX3, NOX4, and NOX5. NADPH is reduced after being utilized as the substrate of NOX2. Under the catalysis of NOX2, the electron can be transferred to O_2_ from NADPH on the cytosolic side of the phagosomal membrane, thereby resulting in the increase of O_2_^−^. Thus, NOX enzymes are the major source of ROS (Spencer and Engelhardt, 2014). NADPH is consumed during this process as the major antioxidant factor in cells and can reduce H_2_O_2_ to promote resistance to oxidative stress. Therefore, excessive glucose intake leads to the generation and accumulation of ROS (Han, 2016; Jiang et al., 2018), which can induce pathological oxidative damage in many tissues and cause the retrogradation of redox signaling in cells (Murphy, 2008). Oxygen free radicals, a type of ROS, are chemical species with an unpaired electron produced from molecular oxygen (Turrens, 2003). Both endogenous and exogenous free radicals can negatively impact bioactive factors such as nucleic acids, lipids, and proteins by altering the normal redox status, thus increasing oxidative stress. Free radical-dependent oxidative stress is involved in diabetes (Phaniendra et al., 2015).

**FIGURE 1:**
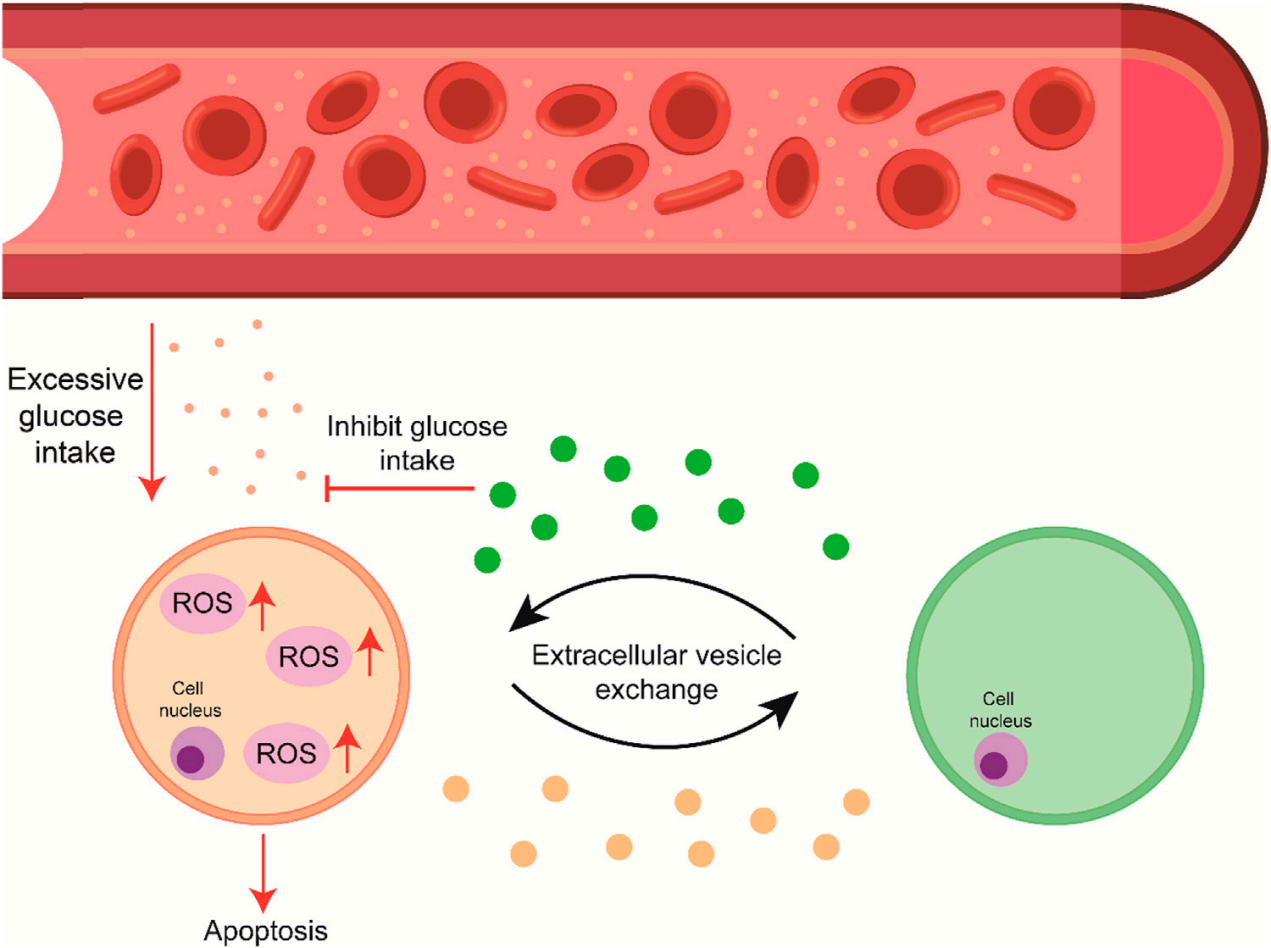
High blood glucose levels lead to ROS generation and accumulation, and the signal exchange through the cell–EV–cell axis promotes cell survival by suppressing apoptosis.

The function of the cell membrane depends on the fluidity and physical state of the membrane, which is determined by the membrane lipid acyl chain profile. The acyl chains of lipids can be peroxidized by ROS, thereby reducing the fluidity of the cell membrane and leading to abnormal membrane function (Watanabe et al., 1990; Eze, 1992). ROS interferes with the normal physiological activities of cells and leads to the decreased expression of insulin-related genes and proteins, such as PDX-1 and MafA (Matsuoka et al., 1997; Bensellam et al., 2012). Hence, in the case of excessive glucose intake-induced insulin resistance, the cells must have a strong requirement to reduce the absorption of glucose to protect the normal function of the membrane (Ye, 2013). Insulin resistance is the hallmark of T2DM, associated with obesity induced by excessive energy intake (Veech, 2004). At the molecular level, insulin resistance is a complex pathological condition consisting of serious pathological phenomena, such as suppressed insulin receptor (IR), down-regulated p-AKT, or up-regulated p-s-IRS (Tanti and Jager, 2009; Tonks et al., 2013). It has been widely accepted that excessive generation and accumulation of ROS will significantly induce insulin resistance (Houstis et al., 2006). High uric acid-induced ROS can significantly inhibit the phosphorylation of AKT, promote the activation of p-s-IRS, and stimulate insulin resistance in differentiated 3T3L1 adipocytes and mice (Zhu et al., 2014; D'Apolito et al., 2010). Nonetheless, the mechanism of how ROS blocks insulin signaling is still unclear. Recent studies have demonstrated that ROS is an induction factor for EV secretion (Hedlund et al., 2011; Aswad et al., 2014; Atienzar-Aroca et al., 2016). O_2_^−^ and H_2_O_2_ are two major stimulators of cell damage. EV secretion promotes a significant increase against H_2_O_2_-induced stress in Jurkat and Raji cells (Hedlund et al., 2011). Therefore, ROS may induce insulin resistance and stimulate the secretion of EVs in a feedback manner (Figure 1).

### EVs form a transport axis between cells to spread insulin resistance

It has been shown that cells will form a signal axis through EVs due to stress-induced cellular damage. Donor cells can secrete EVs in response to the signaling of EVs from recipient cells (Crewe et al., 2018). In response to the signals received from EVs, endothelial cells secrete Caveolin1-containing EVs that are believed to be transported to specific adipocytes lacking the Caveolin1 protein. Furthermore, the process of Caveolin1 transfer relies mainly on EV transmission. When GW4869 inhibits EV secretion, the transmission of Caveolin1 can be significantly reduced (Crewe et al., 2018). However, a similar EV-dependent signaling axis can be formed under a state of excessive glucose intake. Excessive glucose not only promotes the generation and accumulation of ROS but also leads to the synthesis of lipids in cells (Parekh and Anania, 2007), thereby enhancing the secretion and transportation of adiponectin. Moreover, fatty acid-induced adiponectin transportation can be transmitted *via* EVs (DeClercq et al., 2015). Adiponectin is an adipokine involved in regulating the balance of glucose and lipid metabolism. It can reduce the oxidized LDL-induced ROS in endothelial cells (Ouedraogo et al., 2006; Tao et al., 2014). It has been demonstrated that exosomes with abundant adiponectin promote the release of interleukin-6 (IL-6) and tumor necrosis factor-α (TNF-α) from adipocytes (Zhang et al., 2016). IL-6 can regulate the inflammatory response and impair insulin signaling. TNF-α is a pro-inflammatory cytokine involved in systemic inflammation. TNF-α gene knockout mice have a higher sensitivity to insulin in an obese state (Hotamisligil, 1999). The secretion of IL-6 and TNF-α will reduce the translocation of IRS-1 and GLUT4, thus leading to insulin resistance (Uysal et al., 1997; Rotter et al., 2003). Furthermore, adipocyte-derived EVs have been proven to be the major immunomodulatory effectors for the secretion of insulin resistance factors (Kranendonk et al., 2014). Thus, excessive glucose-induced ROS may activate a cell–EV–cell signal axis to help cells under ROS stress gain insulin resistance factors.

### EVs play a critical role in the development of diabetic complications such as DPN

evere complications often accompany diabetes, and neuropathy is the most common (Feldman et al., 2019), leading to many patient deaths. The treatment of DPN can significantly improve patient quality of life. The mechanism of DPN progression is associated with a variety of signaling pathways. Recent studies have shown that EVs have a significant influence on DPN, either positive or negative. Mesenchymal stromal cell (MSC)-derived exosomes significantly increased nerve conduction and inhibited the Toll-like receptor (TLR)4/NF-κB signaling pathway in diabetic mice with DPN (Fan et al., 2020). Exosomes enriched with miR-146a enhanced the therapeutic efficacy of DPN in diabetic mice (Fan et al., 2021). However, as we discussed previously, the function of EVs depends on the cargo they carry. An example is provided by the interaction of Schwann cells with nerve cells. Schwann cells significantly impact nerve cells, such as helping axons form typical large-caliber axons *via* controlling the number of neurofilaments and elevating the phosphorylation state of neurofilaments. EVs mediate intercellular communication between Schwann cells and nerve cells by exchanging their biomaterials. Exosomes derived from high-glucose-stimulated Schwann cells contain high levels of miR-28, miR-31a, and miR-130a, which may contribute to the development of DPN (Jia et al., 2018). Thus, nerve cells may also form a cell–EV–cell axis in response to the stimulation of multiple physiological changes.

## Factors inhibiting insulin signaling can be transmitted *via* EVs

### HFD-induced insulin resistance is due to down-regulated p-y-IRS and up-regulated p-s-IRS

In addition to obesity and other causes, at the molecular level, the abnormality of key proteins in the insulin signal pathway may affect cell sensitivity to insulin, such as the decrease in phosphorylated AKT, the up-regulation of phosphorylated IRS at the serine site (p-s-IRS), and the down-regulation of phosphorylated insulin receptor substrate at the tyrosine site (p-y-IRS) (Gao et al., 2002). Under normal circumstances, a cascade of reactions is activated after insulin binds to its cell surface receptors and causes receptor autophosphorylation. Phosphorylated insulin receptors will recruit their corresponding substrates to accomplish the phosphorylation at the tyrosine site, thereby further activating PI3K and leading to the phosphorylation of AKT. In contrast, p-s-IRS can activate subsequent signal pathways and inhibit p-y-IRS, thereby resulting in insulin resistance (Zhu et al., 2011). Multiple factors for inducing p-s-IRS can result in the increase of free fatty acids, cytokines, angiotensin II, endothelin-1, amino acids, cellular stress, and hyperinsulinemia (Gual et al., 2005). In addition, p-s-IRS can promote a decrease in tyrosine kinase activity (Schmelzle et al., 2006). Recent studies have shown that EVs can transmit these factors that block the insulin signal pathway between cells (Kapogiannis et al., 2015).

### Ubiquitinated IR and IRS packed into EVs can be released into the extracellular environment

**FIGURE 2:**
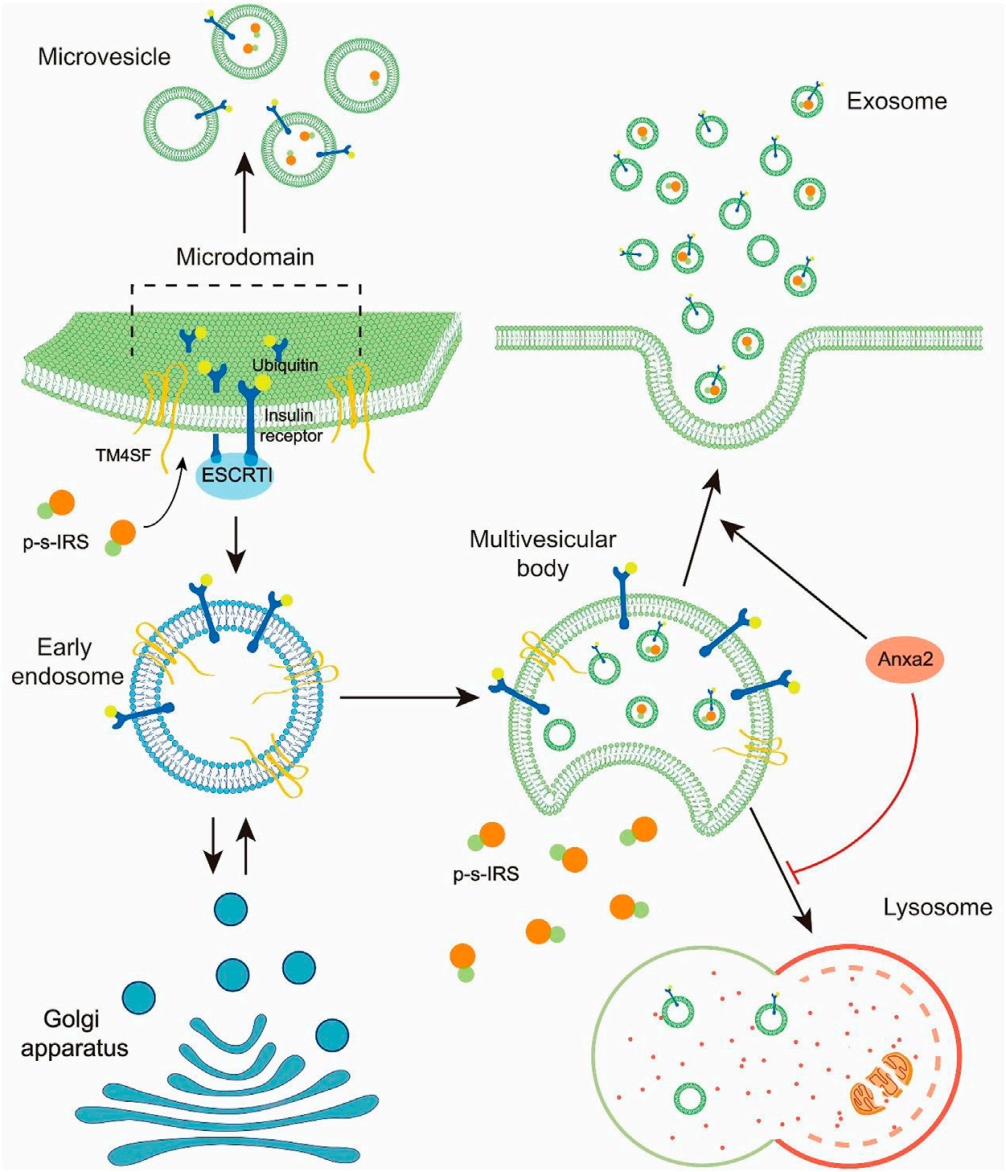
Diagram depicts that p-s-IRS is selectively loaded into EVs and released to the extracellular environment.

Many cells secrete EVs in an evolutionarily conserved manner. There is a wide range of EVs, including classical exosomes, nonclassical exosomes, classical microvesicles, large oncosomes, apoptotic vesicles, and autophagic extracellular vesicles (Jeppesen et al., 2019). Although the biogenesis of microvesicles and exosomes involves different pathways, they have similar morphology, compositions, and functions (Van Niel et al., 2018). As the medium for information transfers between cells, EVs secreted by different cells carry different substances and have multiple targets to cause different effects on recipient cells. Exosomes secreted by renal carcinoma cells will spread to other renal carcinoma cells and eventually cause resistance to sunitinib *via* transporting lncARSR (Qu et al., 2016). However, exosomes containing inflammasomes after central nervous system (CNS) injury can execute the protection of CNS from injury by activating the innate immune response of peripheral tissue (de Rivero Vaccari et al., 2016). Thus, to explore the functions of EVs, the substances in EVs must be studied. Exosomes secreted by T2DM patients have been found to contain p-s-IRS acting as an inhibitor to the insulin signal pathway (Kapogiannis et al., 2015). The most immediate factor for inducing EV formation is the abnormal expression or modification of ubiquitinated proteins. Recent studies have shown that the phosphorylation of IRS-1 at the serine site can lead to its degradation, followed by ubiquitination (Kim et al., 2012; Yoneyama et al., 2018). However, proteins monoubiquitinated on the cell surface are often transferred to multivesicular bodies (MVBs) (Buschow et al., 2005; Gual et al., 2005). Sorting machineries, such as transmembrane proteins and the endosomal sorting complex required for transport (ESCRT), can generate both microvesicles and exosomes (Akers et al., 2013). Exosomes are present in MVBs as intraluminal vesicles (ILVs) before release into the extracellular environment (Kowal et al., 2014; Wang et al., 2020). The MVBs from the early-stage endosomes and the formation of ILVs are involved in specific sorting machineries. These sorting machineries can separate the cargoes into a specific area of the MVE as the microdomain and germinate small membrane vesicles containing isolated cargoes (Kalra et al., 2016). The ESCRT complex is the driver of membrane invagination and budding to accomplish exosome formation in a certain order (Meldolesi, 2018). The ubiquitinated transmembrane cargoes are confined to the microdomain of the MVE by ESCRT-0 and ESCRT-I. Then, the ESCRT-III complex is recruited by ESCRT-II to conduct microdomain formation (Hurley, 2008). Transmembrane proteins are involved in sorting ESCRT-dependent and ESCRT-independent vesicle contents, such as transmembrane 4 superfamily (TM4SF) (Gual et al., 2005). When MVBs are formed, some are transported to lysosomes for degradation rather than fusion with the plasma membrane (Davies et al., 2009). As a calcium-dependent phospholipid-binding protein, Annexin A2 (Anxa2) can be involved in diverse cellular processes. Anxa2-containing MVBs can fuse directly with plasma membranes rather than be degraded by lysosomes (Valapala and Vishwanatha, 2011), and AnxA2 is highly expressed in diabetic patients (Bin et al., 2012). IR and IRS labeled with ubiquitin may be transported to MVBs rather than be degraded (Song et al., 2013; Zhao et al., 2018) (Figure 2). Thus, obesity-induced p-s-IRS or p-s-IR may be released to the extracellular microenvironment by EVs, which makes insulin resistance a transmissible pathological condition.

## The transmission of insulin resistance can be prevented by altered EV cargoes induced by exercise and meditation

**FIGURE 3:**
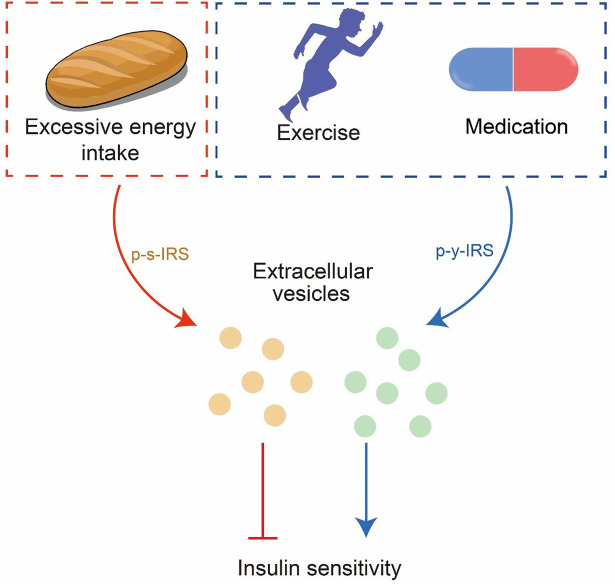
EVs are the information carriers between cells. Excessive energy intake will lead to insulin resistance transmitted through EVs; exercise and meditation can reverse insulin resistance.

T2DM is a metabolic disease caused by excessive energy intake-induced obesity and insulin resistance. It is widely accepted that exercise can promote body weight loss by accelerating excessive energy consumption and increasing the insulin sensitivity of cells (Kjøbsted et al., 2017). However, exercise also can accomplish health promotion or disease rehabilitation *via* EVs (Bertoldi et al., 2018). Multiple tissues release EVs following exercise (D’Souza et al., 2018), and exercise can up-regulate p-y-IRS (Heled et al., 2003; Wrann et al., 2013). Recent studies have demonstrated a significant increase in the amount of EVs during exercise, which restores to pre-exercise level after 4 h. Moreover, EVs induced by exercise tend to be transported to the liver (Whitham et al., 2018; Li et al., 2022). Insulin resistance in the liver is a critical inducement of T2DM (Perry et al., 2014), and pharmacological intervention of glucose metabolism in the liver is an important treatment strategy for T2DM (Lin et al., 2000; Shaw et al., 2005). Hyperinsulinemia is a common symptom of T2DM, leading to decreased insulin signaling in the liver and skeletal muscle by increasing the p-s-IRS level, thereby resulting in insulin resistance (Ueno et al., 2005). Exercise can reduce serum insulin, improve p-y-IRS, and reduce p-s-IRS (Ngo et al., 2002; Heled et al., 2003; Ropelle et al., 2006). Furthermore, exercise-derived exosomes can improve the symptoms of T2DM (Houmard et al., 2004; Safdar et al., 2016). Thus, exercise may block the EV-dependent transmission of insulin resistance and reverse its spread. Similarly, pioglitazone (PIO), a common T2DM drug, can reverse insulin resistance by altering exosome cargo compositions (Kubota et al., 2006; Lopez and Pratley, 2018). The level of p-y-IRS can be improved upon PIO treatment (Hammarstedt et al., 2005), with a similar effect as exercise intervention (Figure 3).

While there has been increased research on EV-related NCD, it is important to note that different sample types have been used in various studies, as summarized in Table 1. As indicated in Table 1, most studies have used either cells or animal samples. Fewer studies have used clinical samples, highlighting the need for greater efforts to analyze clinical samples in future work.

**TABLE 1:** Summary of EV-related NCD studies and their sources of experimental samples.

Source	Reference
Cell	Wang et al. (2017); Ying et al. (2017); Choi et al. (2015); Kowal et al. (2016); Zhu et al. (2016); Jeppesen et al. (2019); Hedlund et al. (2011); Aswad et al. (2014); Atienzar-Aroca et al. (2016); Crewe et al. (2018); DeClercq et al. (2015); Qu et al. (2016); Buschow et al. (2005); Valapala and Vishwanatha (2011); Keryer-Bibens et al. (2006)
Animal	Ying et al. (2017); Choi et al. (2015); Deng et al. (2009); Aswad et al. (2014); Crewe et al. (2018); Qu et al. (2016); Bertoldi et al. (2018); Whitham et al. (2018); Eguchi et al. (2016)
Clinical	Kapogiannis et al. (2015); Kowal et al. (2016); Jeppesen et al. (2019); de Rivero Vaccari et al. (2016); Akers et al. (2013); Wang et al. (2020); D’Souza et al. (2018); Eguchi et al. (2016)

## Conclusion

The current studies on the development of insulin resistance have mainly focused on rescuing insulin resistance rather than suppressing its transmission in the body. Although studies on EVs for regulating the development and progression of insulin resistance have been initiated, the accurate regulatory roles of EVs in the transmission of insulin resistance and underlying mechanisms are still unclear. EVs, the information-exchanging carriers between cells, are involved in multiple pathological signal pathways. Exploring the regulatory roles of EVs in the development and progression of insulin resistance can not only help us understand the mechanisms for blocking the transmission of insulin signaling but also provide us with potentially effective EV-based preventive and therapeutic strategies. However, the functions of EVs depend on their compositions, such as p-s-IRS and p-y-IRS; therefore, exercise or medication interventions may reverse insulin resistance by blocking the transmission of insulin signaling by altering the cargoes of EVs (Figure 3).
